# Inhibition of diacylglycerol O-acyltransferase 1 provides neuroprotection by inhibiting ferroptosis in ischemic stroke

**DOI:** 10.1186/s10020-025-01255-w

**Published:** 2025-05-15

**Authors:** Youjie Zeng, Ren Guo, Songhua Chen, Yuxin Lin, Si Cao, Xia Wang, Siyi Zhang, Huilin Xu, Wenxiang Qing, Heng Yang, Wen Ouyang

**Affiliations:** 1 Department of Anesthesiology, Third Xiangya Hospital, Central South University, 138 Tongzipo Road, Yuelu District, Changsha, 410013 Hunan China; 2 Department of Pharmacy, Third Xiangya Hospital, Central South University, Changsha, 410013 Hunan China; 3 Department of Laboratory Medicine, Third Xiangya Hospital, Central South University, Changsha, 410013 Hunan China; 4 Department of Neurology, Third Xiangya Hospital, Central South University, Changsha, 410013 Hunan China

**Keywords:** DGAT1, Lipid metabolism, Mitochondrial dysfunction, Ferroptosis, Ischemic stroke

## Abstract

**Background:**

Diacylglycerol O-acyltransferase 1 (DGAT1) is crucial for triglyceride synthesis, yet its role in ischemic stroke remains unclear. This study investigated DGAT1 in ischemic stroke using middle cerebral artery occlusion (MCAO) rat models and highly differentiated PC12 cells subjected to oxygen–glucose deprivation/reoxygenation (OGD/R).

**Methods:**

The therapeutic effects of DGAT1 inhibition in MCAO rats were assessed using the Zea-Longa score and 2,3,5-Triphenyltetrazolium chloride (TTC) staining. The effects on highly differentiated PC12 cells subjected to OGD/R were evaluated using the Cell Counting Kit-8 (CCK-8) and lactate dehydrogenase (LDH) assays. Ferroptosis-related mitochondrial damage was evaluated using transmission electron microscope. Additionally, the mechanisms by which DGAT1 inhibition regulates ferroptosis were further explored via immunohistochemistry, immunofluorescence, Western blotting, qPCR, JC-1 assay, and reactive oxygen species (ROS) detection.

**Results:**

DGAT1 expression was elevated in both MCAO and OGD/R models. The DGAT1 inhibitor A 922500 improved neurological deficits, reduced infarct volume, and minimized neuronal loss in MCAO rats, while also enhancing cell viability and reducing LDH levels in OGD/R-treated PC12 cells. DGAT1 inhibition significantly alleviated ferroptosis in MCAO rats, as indicated by (i) reduced mitochondrial shortening and cristae disruption, (ii) decreased 4-HNE levels, (iii) reduced MDA and increased SOD, and (iv) lowered levels of inflammatory factors (IL-6, MCP-1, and TNF-α). Moreover, both in vivo and in vitro experiments showed that DGAT1 inhibition significantly increased Gpx4 levels, whereas lentiviral delivery of Gpx4 shRNA markedly reversed its beneficial effects. In MCAO rats, Gpx4 shRNA significantly elevated 4-HNE levels and exacerbated ferroptosis-related mitochondrial damage. In vitro, DGAT1 inhibition increased mitochondrial membrane potential and reduced ROS, whereas rotenone, a mitochondrial function inhibitor, decreased Gpx4 and impaired cell viability. Furthermore, DGAT1 inhibition significantly upregulated the key β-oxidation gene *Cpt1a*, whereas etomoxir, a β-oxidation inhibitor, reduced cell viability and mitochondrial membrane potential, increased ROS, and downregulated Gpx4.

**Conclusions:**

Our study suggests that DGAT1 inhibition may enhance β-oxidation and mitochondrial function, thereby increasing Gpx4 levels, suppressing ferroptosis, and ultimately exerting neuroprotective effects in ischemic stroke.

**Supplementary Information:**

The online version contains supplementary material available at 10.1186/s10020-025-01255-w.

## Introduction

Ischemic stroke, a leading cause of disability and mortality worldwide, is characterized by a sudden interruption of cerebral blood flow, resulting in neuronal injury and brain tissue damage due to ischemia and reperfusion (Feske 2021). In 2021, there were an estimated 7.8 million (95% UI 6.7–8.9 million) incident cases of ischemic stroke globally, with an associated mortality rate of 44.2 per 100,000 population (95% UI 39.5–47.8), contributing to a total of 70.4 million (95% UI 64.1–76.0 million) disability-adjusted life years (GBD 2021). In addition, by 2030, the global age-standardized incidence rate of ischemic stroke is expected to increase to 89.32 cases per 100,000 individuals (Pu et al. 2023). Tissue plasminogen activator (tPA) is the only FDA-approved treatment for acute ischemic stroke, but its application is limited by a narrow 3–4.5-h therapeutic window (Institute and of Neurological D, Stroke rt PASSG. 1995; Adibhatla and Hatcher 2008; Henninger and Fisher 2016; Lansberg et al. 2009). Endovascular thrombectomy improves outcomes in cases of large vessel occlusion but increases the risk of intracranial hemorrhage (Goyal et al. 2016). Thus, ongoing research into the molecular mechanisms and neuroprotective therapies remains crucial for improving ischemic stroke outcomes.

Among all human tissues, the brain has the second-highest lipid content, following adipose tissue, with lipids comprising approximately 50% of its dry weight (Yoon, et al. 2022). Recent studies have highlighted that disturbances in lipid metabolism are a key pathological feature of ischemic stroke, contributing to neuronal damage by disrupting cellular homeostasis during ischemia and reperfusion (Todorović, et al. 2021). As a key enzyme in triacylglycerol (TAG) biosynthesis and a regulator of lipid droplet formation, diacylglycerol O-acyltransferase 1 (DGAT1) plays a crucial role in cellular lipid storage, supply, and homeostasis (Chitraju et al. 2017). DGAT1 catalyzes the final step by converting diacylglycerol (DAG) and acyl-CoA into TAG (Cases et al. 1998). Previous studies targeting DGAT1 across various cancers have yielded conflicting results regarding its effects on tumor progression (Mitra et al. 2017; Nardi et al. 2019; Cheng et al. 2020a; Cheng et al. 2020b; He et al. 2021; Morales et al. 2021; Xia et al. 2021; Wilcock et al. 2022; Zhou et al. 2022; Liu et al. 2022a; Kang et al. 2023; Cui et al. 2024; Deng et al. 2024; Deskeuvre et al. 2024; Ghimire 2024; Wang et al. 2024). Despite the potential of targeting metabolic pathways as a therapeutic strategy for ischemic stroke, the specific role of DGAT1 in this context remains unclear (Liang et al. 2021).

Ferroptosis is a recently identified, non-apoptotic form of programmed cell death, characterized by mitochondrial fragmentation, increased membrane density, reduced or absent cristae, and accumulation of lipid reactive oxygen species (ROS) (Li et al. 2020). In the context of ischemic stroke, the relationship between ferroptosis and neuronal injury has garnered significant attention. During ischemic events, mitochondrial dysfunction is prevalent, leading to increased production of ROS (Tian, et al. 2023). This oxidative stress not only contributes to neuronal damage but also accelerates lipid peroxidation—a critical step in the initiation of ferroptosis (She et al. 2023). Research indicates that inhibition of ferroptosis can mitigate ischemic injury; for instance, the ferroptosis-specific inhibitor ferrostatin-1 has been shown to reduce damage in middle cerebral artery occlusion (MCAO) rat models (Liu et al. 2023a). Targeting ferroptosis may offer a promising strategy to alleviate neuronal injury in ischemic stroke (Wei et al. 2022). Given that DGAT1 regulates lipid metabolism, and that disruptions in lipid metabolism are linked to mitochondrial dysfunction—a key contributor to ferroptosis—we aim to investigate whether DGAT1 influences ischemic stroke progression by modulating lipid homeostasis, mitochondrial function, and ferroptosis.

In this study, we first examined DGAT1 expression in both in vivo MCAO rat models and in vitro oxygen–glucose deprivation/reperfusion (OGD/R) models. We further investigated the effects of the DGAT1-specific inhibitor A 922500 on post-hypoxic injury phenotypes, as well as its impact on ferroptosis, mitochondrial function, and lipid metabolism in both in vivo and in vitro settings. Overall, this study provides preliminary insights into DGAT1 as a potential therapeutic target for ischemic stroke.

## Materials and methods

### Gene expression omnibus microarray dataset analysis

GSE97537 dataset from the Gene Expression Omnibus (GEO) database includes brain tissue samples from 7 Sprague–Dawley (SD) rats subjected to the MCAO model and 5 SD rats from the sham-operated group. The gene expression matrix was generated by reading the CEL files and performing normalization using the “oligo” R package. Gene probes were then converted into gene symbols using the “rat2302.db” R package, and the DGAT1 expression levels were extracted from all samples. Based on the results of the normality test, either an independent-samples t-test or a Mann–Whitney U test was used to assess DGAT1 expression differences between the MCAO and sham groups.

### Animals

The in vivo animal study was approved by the Laboratory Animal Welfare Ethics Committee of Central South University. All rats were housed under specific pathogen-free (SPF) conditions with a 12-h light/dark cycle and were provided free access to food and water. Six animals were ultimately included in each group for each experiment. A random number table was used to assign animals to groups in a randomized order. All post-surgical procedures, including neurological scoring, sample collection, and other experimental assessments, were performed by personnel who were blinded to the group assignments and not involved in the model construction, in order to ensure blinding. Inclusion criteria included male Sprague–Dawley rats weighing 250–300 g, SPF grade, and good general health. For the MCAO group, animals were additionally required to have a Longa neurological score ≥ 1. Exclusion criteria included anatomical anomalies preventing proper insertion of the silicone-coated suture, severe postoperative infection, death within 24 h after surgery, or visible cerebral hemorrhage.

### Intracerebroventricular injection

After 12 h of fasting, rats were anesthetized with sodium pentobarbital (50 mg/kg) administered via intraperitoneal injection. Next, the rats were placed in the prone position on a stereotaxic apparatus (Stoelting Co. Ltd, USA) with the head securely fixed. Following preparation and disinfection of the scalp, a midline incision was made, and the subcutaneous tissue and periosteum were separated to expose the bregma. A cranial hole was drilled 1.5 mm lateral to the right and 1 mm posterior to the bregma. A microsyringe was inserted to a depth of 4 mm, and 10 μL of A 922500 (50 μmol/L) was injected into the lateral ventricle at a rate of 1 μL/min using a microinjection pump. Following injection, the syringe was left in place for 10 min to ensure adequate absorption before being slowly withdrawn. The cranial hole was sealed with bone wax, and the scalp was sutured.

### Construction of MCAO rat models

MCAO models were established 2 h after intracerebroventricular injection. The procedure was performed according to the method previously described by Longa et al. (1989). Specifically, following neck preparation and disinfection, a midline cervical incision was made. Blunt dissection exposed the right common carotid artery (CCA), internal carotid artery (ICA), and external carotid artery (ECA). The CCA and ICA were clamped, the distal ECA ligated and severed, and a small incision was made in the proximal ECA. A silicone-coated suture was inserted and advanced into the ICA until slight resistance was felt, indicating occlusion of the right middle cerebral artery. After 2 h of occlusion, the suture was gently withdrawn to allow reperfusion.

### Immunohistochemistry (IHC) staining

Fresh brain tissue is fixed in 4% paraformaldehyde for over 24 h, trimmed, and placed in embedding cassettes. After a 20-min water wash, tissues were dehydrated in graded ethanol (75%, 85%, 95%, absolute), cleared in xylene, and embedded in paraffin. Once solidified, paraffin blocks were sectioned into 4 µm-thick coronal slices, mounted onto slides, and dried at 60 °C for 30 min to 2 h. For immunohistochemistry, sections were dewaxed, rehydrated, and antigen retrieval was performed using EDTA (pH 9.0) or citrate buffer (pH 6.0) in a pressure cooker or microwave. Endogenous peroxidase activity was blocked with 3% H_2_O_2_, followed by blocking with 10% serum for 30 min. Sections were incubated with primary antibodies overnight at 4 °C, followed by secondary antibody incubation at 37 °C for 45 min. DAB staining was then performed, followed by hematoxylin counterstaining. Finally, slides were mounted and examined under a microscope. The primary antibodies used were DGAT1 (Proteintech, Cat. No. 11561–1-AP) and 4-HNE (Bioss Antibodies, Cat. No. bs-6313R).

### Immunofluorescence staining and co-localization

Tyramide Signal Amplification (TSA) enhances fluorescence signals through HRP-catalyzed deposition of activated tyramide at antigen sites, which binds covalently to tyrosine residues, enabling for significant signal amplification and stable labeling. For double- label multiplex immunofluorescence, standard dewaxing and rehydration steps were performed as described in the IHC protocol. Antigen retrieval was conducted using EDTA (pH 9.0) or citrate buffer (pH 6.0) in a microwave (8 min at medium power, 8 min rest, 7 min at medium–low power), followed by PBS washing. Endogenous peroxidase was blocked for 15 min, followed by BSA blocking for 60 min at 37 °C. Sections were incubated with the first primary antibody, DGAT1 (Proteintech, Cat. No. 11561–1-AP), overnight at 4 °C. After washing, an HRP-conjugated secondary antibody was applied, followed by TSA-520 incubation for 10–15 min. Antigen retrieval and blocking steps were repeated for the second primary antibody, NeuN (Abiowell, Cat. No. AWA10318), followed by incubation with the corresponding secondary antibody and TSA-570. Finally, nuclei were counterstained with DAPI, and sections were mounted and imaged for DGAT1 and NeuN co-localization under a fluorescence or confocal microscope.

### Cell culture and construction of OGD/R models

The highly differentiated PC12 cells (Abiowell, Cat. No. AW-CCR070) were used for in vitro experiments. This cell line is derived from transplantable pheochromocytoma of the male rat adrenal medulla. These cells express nerve growth factor (NGF) receptors, and NGF can induce the development of a neuronal phenotype. The cells were cultured in RPMI-1640 (Gibco, Cat. No. 11875093) containing 10% fetal bovine serum (FBS; Procell, Cat. No. 164210–50) and 1% penicillin/streptomycin (Solarbio, Cat. No. P1400) at 37 °C in an incubator with 5% CO_2_ and 95% air.

Sample size determination, randomization, and blinding procedures were consistent with those used in the animal experiments. PC12 cells were required to meet the following inclusion criteria prior to any procedure: (1) cells exhibited stable neuron-like morphology; (2) cultures were free of contamination; (3) cell confluence was ≥ 60% as assessed by visual inspection under an inverted microscope; and (4) cells had undergone at least three passages to ensure stability.

The method for establishing the OGD/R model was as follows: under the original culture conditions for PC12 cells, 10% FBS was omitted, and RPMI-1640 was replaced with glucose-free DMEM (Gibco, Cat no: 11966025). The cells were then placed in an incubator with 95% N₂ and 5% CO₂ at 37 °C for 6 h. Subsequently, the cells were cultured under the original normal conditions for 24 h for re-oxygenation. All experimental interventions on PC12 cells were conducted simultaneously during the initial phase of establishing the OGD/R model.

### Western blotting assay

Total protein was extracted from highly differentiated PC12 cells and rat brain tissue using RIPA lysis buffer (Biosharp, Cat. No. BL504 A). Protein concentration was determined using the Bicinchoninic Acid (BCA) protein assay kit (Biosharp, Cat. No. BL521 A). Samples were combined with loading buffer (Biosharp, Cat. No. BL502 A) in the proper ratio, heated at 95 °C for 10 min for denaturation, and subsequently stored at − 40 °C. Then, equal amounts of protein (50 μg) from each sample were resolved by 12% SDS-PAGE and transferred onto a polyvinylidene fluoride (PVDF) membrane. The PVDF membranes were then blocked with 5% skim milk at 37 °C for 1 h, followed by incubation with the corresponding primary antibody overnight at 4 °C: anti-DGAT1 (Proteintech, Cat. No. 11561–1-AP), anti-FSP1 (ABclonal, Cat. No. A22278), anti-GPX4 (Abiowell, Cat. No. AWA11352), anti-DRP1 (Abiowell, Cat. No. AWA00145), anti-MFN2 (Abiowell, Cat. No. AWA41852), anti-β-actin (Proteintech, Cat. No. 20536–1-AP). The membranes were then treated with an HRP-conjugated secondary antibody at 37 °C for 1 h. Chemiluminescent signals were detected using the ECL chemiluminescent substrate (Biosharp, Cat. No. BL520 A).

### TTC (2,3,5-triphenyltetrazolium chloride) staining

Twenty-four hours after establishing the MCAO model, the rats were euthanized. The brains were then extracted and sectioned into six equal parts. The sections were stained with 2% 2,3,5-Triphenyltetrazolium chloride (TTC; MedChemExpress, Cat. No. HY-D0714) at 37 °C for 30 min, protected from light. The slices were then fixed by immersion in 4% paraformaldehyde overnight. Infarct volume was calculated using Image J software.

### Neurological function assessed using Longa's score

The Longa score system assesses the severity of neurological deficits by observing and recording the rats'motor and behavioral performance (Longa et al. 1989). A higher score indicates more severe neurological impairment. The score ranges from 0 to 4, with the specific criteria as follows: a score of 0 indicates no neurological deficit, with the rat displaying normal activity; a score of 1 indicates mild neurological deficit, in which the rat can extend its forelimb but cannot fully spread it; a score of 2 indicates moderate deficit, with the rat showing circling movements while walking; a score of 3 indicates severe deficit, where the rat tilts to the side of paralysis; and a score of 4 indicates very severe neurological deficit, in which the rat has completely lost voluntary motor function and shows impaired consciousness.

### Hematoxylin–Eosin (HE) staining

Tissue sections were dewaxed in a clearing agent for 10 min, repeated twice, followed by rehydration through progressively diluted ethanol (100%, 95%, 85%, 75%, each for 5 min) and rinsed with distilled water for 1 min. Sections were then stained with Harris hematoxylin (Biossci, Cat. No. BP0211) for 4 min, rinsed in tap water for 2 min, and differentiated with 0.8% hydrochloric acid alcohol for 2 s. After washing, sections were optionally blued with lithium carbonate and rinsed for 2 min. Eosin (Biossci, Cat. No. BP0211) staining followed for 20 s, with sections dehydrated in 95% ethanol for 5 s and absolute ethanol I and II (2 min each). Finally, sections were cleared with xylene, mounted, and observed under a microscope.

### Nissl staining

Tissue sections were dewaxed in a clearing agent for 10 min (repeated twice) and rehydrated through ethanol gradients (100%, 95%, 85%, 75%, each for 5 min) before rinsing in distilled water for 1 min. The sections were stained with 1% toluidine blue solution (Biossci, Cat. No. BP0360) at 56 °C for 20 min, then washed with distilled water. Differentiation was performed with 95% ethanol or 0.1% acetic acid until Nissl bodies were visible. The sections were dehydrated with absolute ethanol, cleared, mounted with neutral balsam, and examined under a microscope.

### Cell Counting Kit-8 (CCK-8) assay

Highly differentiated PC12 cells were seeded into 96-well plates at a density of 1000–2000 cells per well in 100 μL medium and pre-incubated for 24–48 h. Cells were treated according to different experimental groups and further incubated. Then, 10 μL of CCK-8 solution (APExBIO, Cat. No. K1018) was added to each well, avoiding bubble formation, and the cells were incubated for an additional 1–4 h. The optical density (OD) was measured at 450 nm using a microplate reader, and the results were recorded.

### Lactate dehydrogenase assay

Following treatment of highly differentiated PC12 cells with various interventions, lactate dehydrogenase (LDH) activity in the cell culture supernatant was assessed using an LDH assay kit (Nanjing Jiancheng Bioengineering Institute, Cat. No. A020-2–2, JianCheng Bioengineering Institute, Nanjing, Jiangsu, China) in accordance with the manufacturer's protocol.

### Transmisson electron microscopy

After perfusing rat brain tissue with 4% paraformaldehyde solution, or after obtaining highly differentiated PC12 cell pellets through centrifugation, the samples were fixed with 2.5% glutaraldehyde at 4 °C for 24 h. Then, the glutaraldehyde was discarded, and the samples were fixed in 1% osmium tetroxide solution at room temperature, protected from light, for 2 h. After each fixation step, the samples were rinsed three times with 0.1 M phosphate buffer (PB, pH 7.4). Subsequently, the samples underwent dehydration at room temperature, infiltration, embedding, polymerization, ultrathin sectioning, and staining, followed by observation under a transmission electron microscope (TEM) (Hitachi High-Tech, HT7700).

### Measurement of oxidative stress

Peripheral blood was collected from the apex of SD rats and centrifuged to obtain plasma. The peripheral blood levels of superoxide dismutase (SOD) and malondialdehyde (MDA) were measured using a SOD assay kit (Ruixin Biotech, Cat. No. RXWB0482-96) and an MDA assay kit (Ruixin Biotech, Cat. No. RXFG0005-96), respectively, according to the manufacturer's instructions.

### Measurement of inflammatory factors using Enzyme-Linked Immunosorbent Assay (ELISA)

Plasma levels of IL-6, MCP-1, and TNF-α were measured in six groups using ELISA kits (Ruixinbio, Quanzhou, China) following the manufacturer’s instructions. Briefly, 50 µL of plasma samples or standards were added to 96-well plates pre-coated with specific antibodies for IL-6, MCP-1, or TNF-α. After incubation and washing, HRP-conjugated secondary antibodies were added. The reaction was stopped with 2 M sulfuric acid following substrate addition, and absorbance was measured at 450 nm. Cytokine concentrations were calculated using a standard curve.

### In vitro and in vivo Gpx4 silencing using lentiviral shRNA

To knock down Gpx4 expression, lentiviral vectors encoding short hairpin RNA (Lv-shRNA) specifically targeting Gpx4, as well as a non-targeting shRNA control, were designed and produced by Genechem (Shanghai, China). Based on previous studies and preliminary experimental results, highly differentiated PC12 cells were infected with lentivirus at a multiplicity of infection (MOI) of 20 (Gu et al. 2021). Three different shRNA constructs targeting Gpx4 were designed: LV-955 (sequence: ATGCTGGGAAATGCCATCAAA), LV-956 (sequence: CAGGTTTGACATGTACAGCAA), and LV-957 (sequence: ACGCCGAGTGTGGTTTACGAA). After infection, the stably transduced cells were selected using complete culture medium containing 4 μg/mL puromycin. The knockdown efficiency of the three Gpx4 shRNAs was evaluated by Western blot analysis. The shRNA construct that resulted in the most significant reduction of Gpx4 expression was selected for in vivo experiments.

For the in vivo experiments, the lentivirus encoding LV-956, which showed the highest Gpx4 knockdown efficiency, was selected and injected into the right lateral ventricle at a rate of 1 μL/min, with a total volume of 10 μL (7 × 10^8 TU/ml). Fourteen days after the injection, in vivo experiments and MCAO model construction were performed.

### Measurement of mitochondrial function assessed by JC-1 staining and ROS

Highly differentiated PC12 cells were divided into different groups and cultured in a 96-well plate. The JC-1 working solution was prepared by diluting 50 µL of JC-1 (200 ×) in 8 mL ultrapure water, followed by adding 2 mL of 5 × JC-1 staining buffer (Solarbio, Cat. No. M8650). After washing with PBS, 50 µL of cell culture medium and 50 µL of JC-1 working solution were added to each well for all groups. Cells were incubated at 37 °C for 20 min. Following incubation, the staining solution was removed, and cells were washed twice with 1 × JC-1 staining buffer. According to the manufacturer's instructions, Dihydroethidium (DHE, Beyotime, Cat. No. S0063) was used to measure ROS production in highly differentiated PC12 cells. The highly differentiated PC12 cells were incubated in 5 μM DHE at 37 °C in the dark for 30 min, followed by washing three times with PBS. Fluorescence images for both JC-1 (mitochondrial membrane potential) and DHE (ROS production) were captured using a high-content imaging system. The fluorescence intensity for each was analyzed using ImageJ software.

### RNA extraction and quantitative real-time polymerase chain reaction

Total RNA was extracted from highly differentiated PC12 cells using the Ultrapure RNA Kit (CWBIO), following the manufacturer's protocol. Reverse transcription was carried out to synthesize cDNA using the HiFiScript cDNA Synthesis Kit (CWBIO). Amplification was performed using the ChamQ Universal SYBR qPCR Master Mix (Vazyme) in a quantitative reverse transcription PCR (qRT-PCR) system. β-actin mRNA was used as an internal control for *Cpt1a*, and the relative fold differences were calculated using the 2^−ΔΔCT^ method. The primers, designed and synthesized by Sangon Biotech, were as follows: for *Cpt1a*, the forward primer sequence was 5′-CTGCTGTATCGTCGCACATTAG-3′ and the reverse primer sequence was 5′-CGGGAAGTATTGAAGAGTCGC-3′; for β-actin, the forward primer sequence was 5′-CCCATCTATGAGGGTTACGC-3′ and the reverse primer sequence was 5′-TTTAATGTCACGCACGATTTC-3′.

### Statistical analysis

The data were presented as mean ± standard deviation. Normality was assessed using the Shapiro–Wilk test, and Levene’s test was used to examine the homogeneity of variances. For comparisons between two groups, if the data were normally distributed and variances were equal, an independent-samples t-test was used. If variances were unequal, a Welch’s t-test was applied. For non-normally distributed data, the Mann–Whitney U test was used. For comparisons among more than two groups, if the data met assumptions of normality and homogeneity of variances, a one-way ANOVA followed by Tukey's HSD post-hoc test was used. If variances were unequal, Welch’s ANOVA with Games–Howell post-hoc test was performed. In the case of non-normal data, the Kruskal–Wallis test followed by Dunn’s test with False Discovery Rate correction was used. Statistical analysis and visualization were performed using R software (version 4.3.1). A *p*-value of < 0.05 was considered statistically significant.

## Results

### Inhibition of DGAT1 alleviated neuronal damage caused by MCAO and OGD/R models

**Fig. 1:**
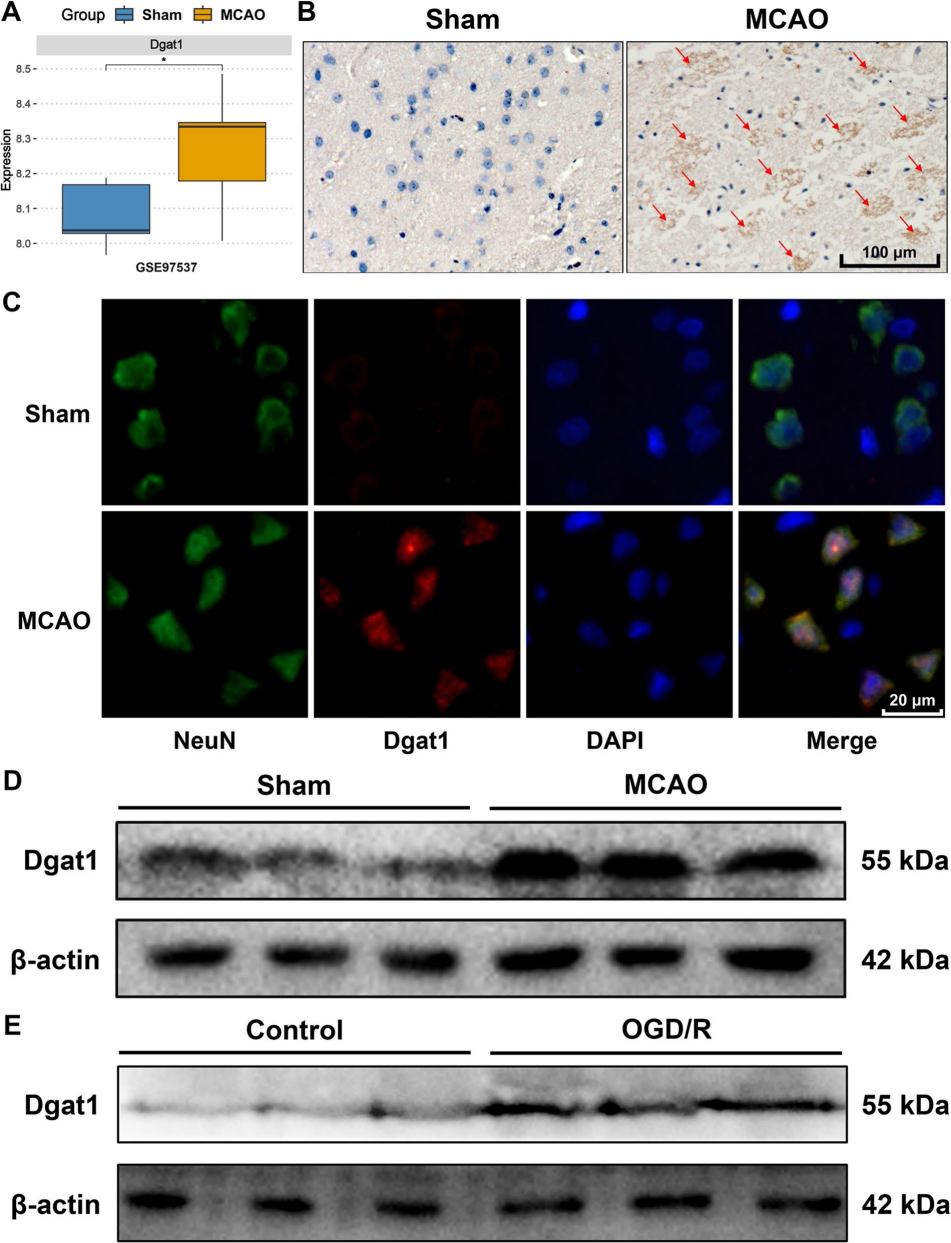
DGAT1 is upregulated in MCAO and OGD/R models. **A** In the GSE97537 dataset, *DGAT1* mRNA levels were significantly upregulated in MCAO rat models compared to the sham group. **B** IHC analysis showing significantly increased DGAT1 expression in the brain tissue of MCAO rat compared to the sham group. **C** Fluorescence co-localization analysis demonstrating significant co-expression of DGAT1 with the neuronal marker NeuN, indicating predominant DGAT1 expression in neurons in the MCAO model. **D** Western blot analysis showing significant upregulation of DGAT1 expression in MCAO group compared to the sham controls. **E** Western blot analysis showing significant upregulation of DGAT1 expression in highly differentiated PC12 cells subjected to OGD/R compared to the control group

In the GSE97537 dataset, *DGAT1* mRNA levels were significantly upregulated in the brain tissue of MCAO rats compared to the sham group (Fig. 1A). Similarly, IHC results confirmed that DGAT1 expression was significantly elevated in the brain tissue of MCAO rats compared to the sham controls (Fig. 1B). Furthermore, co-localization analysis showed that DGAT1 was co-expressed with the neuronal marker NeuN, suggesting that DGAT1 might primarily function in neurons in the MCAO model (Fig. 1C). In vivo, western blot analysis showed a significant increase in DGAT1 expression in the MCAO model compared to the sham controls (Fig. 1D). In vitro, western blot analysis revealed that highly differentiated PC12 cells subjected to OGD/R exhibited a significant increase in DGAT1 expression compared to the control group (Fig. 1E). These findings collectively suggest that DGAT1 expression is significantly upregulated in both the MCAO and OGD/R models.

**Fig. 2:**
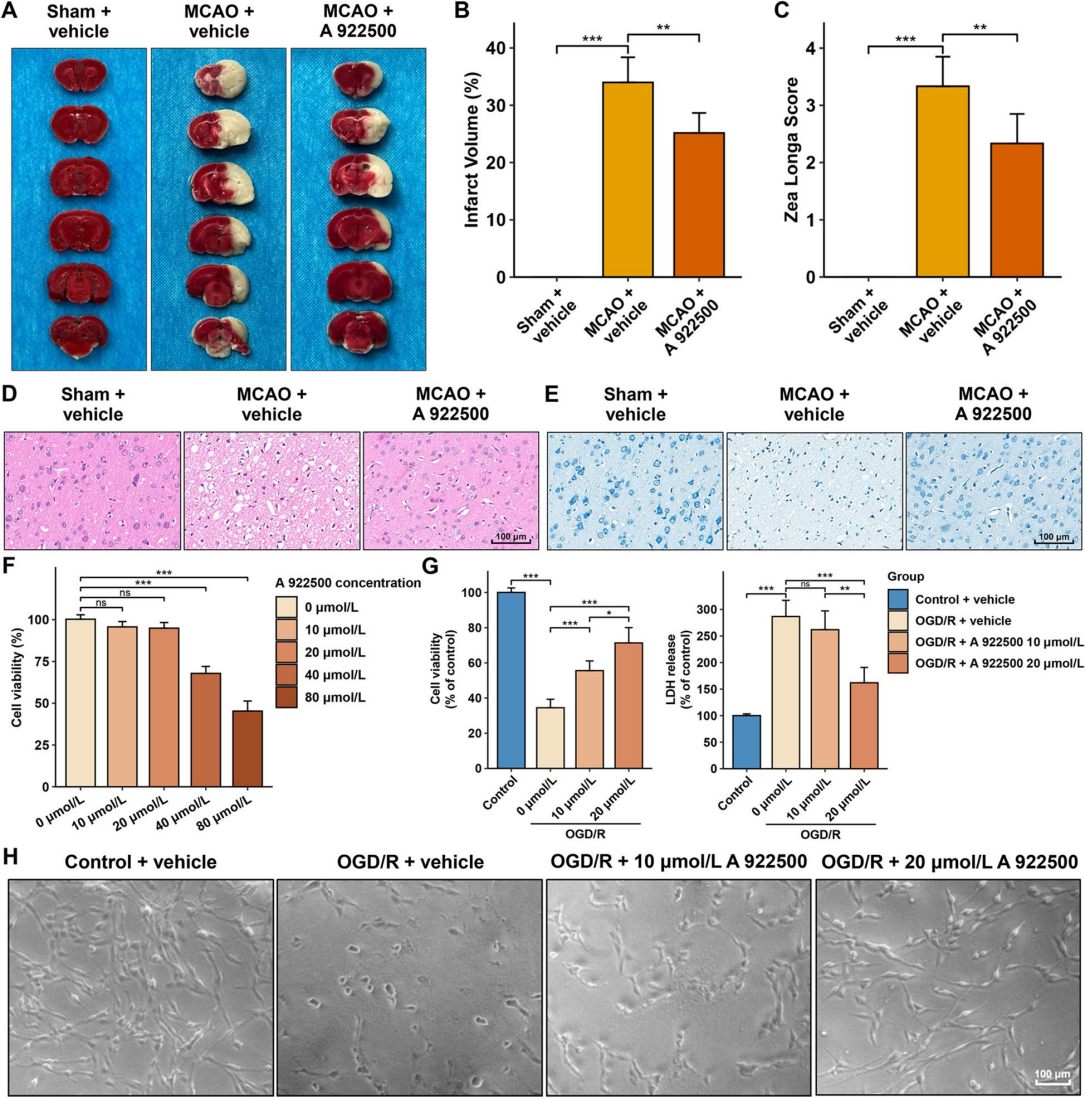
DGAT1 inhibition exerts neuroprotective effects in both in vivo and in vitro models of ischemic stroke. **A** TTC staining showing the effect of DGAT1 inhibition on MCAO-induced infarct volume in rat brains. **B** Quantification of infarct volume in rat brain tissues from different groups based on TTC staining results. **C** Bar plot demonstrating the effect of DGAT1 inhibition on Zea Longa scores in MCAO rats. **D** H&E staining illustrating the improvement in brain pathological damage caused by MCAO following DGAT1 inhibition. **E** Nissl staining showing that DGAT1 inhibition alleviates neuronal damage induced by MCAO. **F** CCK-8 cytotoxicity assay determining the candidate concentrations of the DGAT1 inhibitor A 922500 for in vitro experiments. **G** CCK-8 and LDH assays assessing the protective effects of DGAT1 inhibition on OGD/R-induced injury in highly differentiated PC12 cells. **H** Optical microscopy images showing the effects of DGAT1 inhibition on cell morphology and survival in OGD/R-treated highly differentiated PC12 cells

TTC staining revealed that MCAO significantly increased infarct volume compared to the sham group (Fig. 2A, B). However, treatment with the DGAT1-specific inhibitor A 922500 significantly reduced the infarct volume caused by MCAO (Fig. 2A, B). In addition, DGAT1 inhibition significantly lowered the elevated Zea Longa scores induced by MCAO (Fig. 2C). As shown in Fig. 2D, H&E staining indicated that brain tissue swelling, disorganized cellular arrangement, and cellular deformation were significantly worsened in the MCAO group compared to the sham group. However, after intraventricular injection of the DGAT1-specific inhibitor A 922500, these pathological changes were notably alleviated (Fig. 2D). Furthermore, Nissl staining demonstrated a significant reduction in Nissl body numbers and staining intensity in the MCAO group compared to the sham group, indicating substantial neuronal loss (Fig. 2E). In contrast, inhibition of DGAT1 increased Nissl body numbers and staining intensity, suggesting reduced neuronal loss and restoration of neuronal density and morphology. These findings collectively suggest that inhibition of DGAT1 provides neuroprotection against MCAO-induced brain injury in the rat model.

Subsequently, in vitro experiments were conducted to further verify the protective effects of DGAT1 inhibition on highly differentiated PC12 cells subjected to OGD/R-induced injury. A CCK-8 cytotoxicity assay identified 10 μmol/L and 20 μmol/L as candidate doses for the DGAT1 inhibitor A 922500 (Fig. 2F). The CCK-8 assay further demonstrated that OGD/R significantly reduced the cell viability of highly differentiated PC12 cells. However, treatment with 10 μmol/L and 20 μmol/L of A 922500 significantly increased cell viability in a dose-dependent manner compared to the OGD/R group (Fig. 2G). Similarly, the LDH assay showed that OGD/R significantly increased LDH release, indicating elevated cellular damage. Treatment with A 922500 at both concentrations resulted in a dose-dependent reduction in LDH release, suggesting reduced cellular injury (Fig. 2G). As shown in Fig. 2H, optical microscopy revealed that the control group displayed normal cell morphology with a high number of well-organized cells, whereas the OGD/R group showed significant damage, including cell shrinkage, deformation, disorganization, and a reduced cell count. Treatment with 10 μmol/L A 922500 partially improved cell morphology and increased cell count, while 20 μmol/L A 922500 further restored cell structure and number, demonstrating a dose-dependent protective effect. Based on these findings, 20 μmol/L was selected for subsequent in vitro experiments. These results suggest that DGAT1 inhibition effectively mitigates OGD/R-induced damage in highly differentiated PC12 cells in vitro.

### Inhibition of DGAT1 attenuated ferroptosis in the MCAO model

**Fig. 3:**
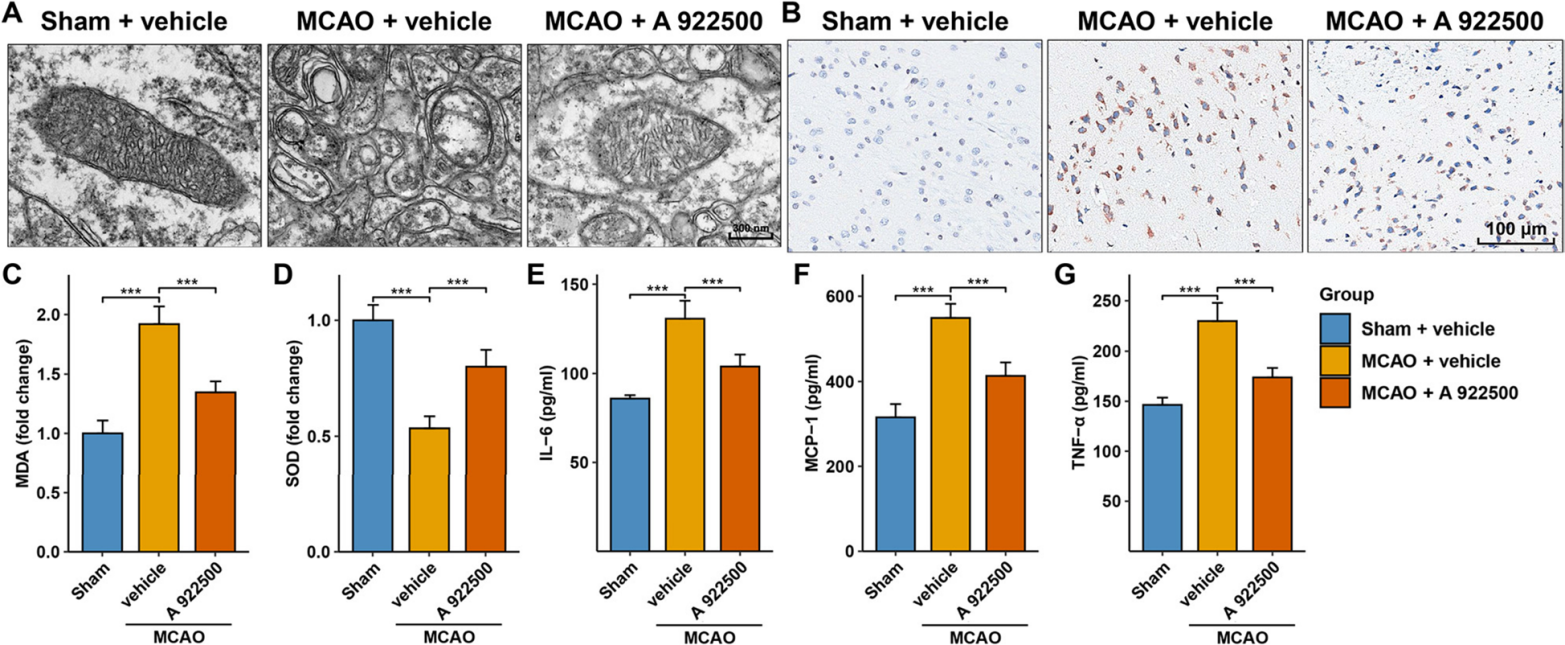
DGAT1 inhibition attenuates ferroptosis in the MCAO model. **A** TEM showing that DGAT1 inhibition improved ferroptosis-associated mitochondrial abnormalities. **B** IHC showing that DGAT1 inhibition alleviated the MCAO-induced upregulation of 4-HNE. **C** Bar plot showing that DGAT1 inhibition reduced the MCAO-induced MDA elevation. **D** Bar plot showing that DGAT1 inhibition reversed the MCAO-induced reduction in SOD levels. **E** Bar plot showing that DGAT1 inhibition reduced the MCAO-induced elevation of IL-6. **F** Bar plot showing that DGAT1 inhibition reduced the MCAO-induced elevation of MCP-1. **G** Bar plot showing that DGAT1 inhibition reduced the MCAO-induced elevation of TNF-α

TEM observations revealed that mitochondria in the sham group exhibited well-preserved structures with distinct cristae, while the MCAO group showed fragmented and impaired cristae, along with shortened mitochondrial length in neurons near the ischemic region (Fig. 3A). However, treatment with the DGAT1 inhibitor A 922500 reduced the extent of mitochondrial cristae damage and rupture, and increased mitochondrial length in the MCAO model (Fig. 3A). Additionally, IHC analysis showed that the MCAO model significantly increased the lipid peroxidation marker 4-HNE compared to the sham group (Fig. 3B). Inhibition of DGAT1 reduced 4-HNE levels compared to the MCAO group (Fig. 3B). Furthermore, MCAO led to a significant increase in MDA levels and a decrease in SOD levels compared to the sham group (Fig. 3C, D). Inhibition of DGAT1 significantly lowered MDA levels and increased SOD levels compared to the MCAO group (Fig. 3C, D). Finally, the levels of three inflammatory factors—IL-6, MCP-1, and TNF-α—were significantly elevated in the MCAO group compared to the sham controls (Fig. 3E, F, G). Inhibition of DGAT1 led to a significant reduction in all three inflammatory factors compared to the MCAO group (Fig. 3E, F, G). These findings indicate that DGAT1 inhibition attenuates ferroptosis in the MCAO model.

### Inhibition of DGAT1 protected highly differentiated PC12 Cells in the OGD/R model by attenuating ferroptosis through upregulating Gpx4

**Fig. 4:**
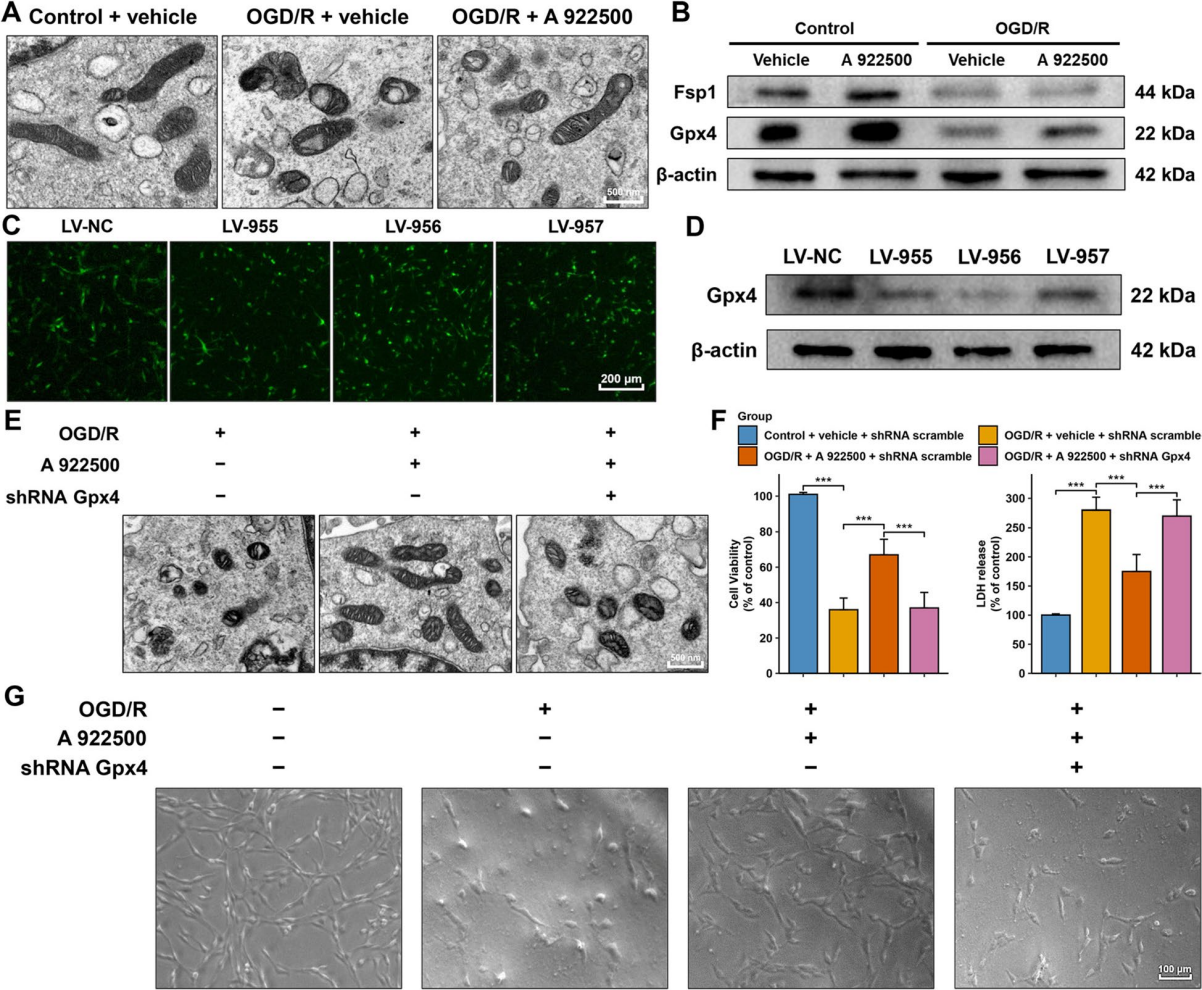
Inhibition of DGAT1 protected highly differentiated PC12 cells in the OGD/R model by attenuating ferroptosis through upregulating Gpx4. **A** TEM images showing that the DGAT1 inhibitor A 922500 alleviated ferroptosis-related mitochondrial damage in highly differentiated PC12 cells following OGD/R treatment. **B** Western blot analysis showing the effect of DGAT1 inhibition on two ferroptosis resistant proteins (Fsp1 and Gpx4). **C** Lentiviral infection carrying Gpx4 shRNA was successfully achieved in highly differentiated PC12 cells. **D** The most effective Gpx4 shRNA sequence was identified by western blot analysis. **E** TEM showing that Gpx4 shRNA significantly reversed the improvements in ferroptosis-related mitochondrial morphology induced by DGAT1 inhibition in OGD/R-treated highly differentiated PC12 cells. **F** CCK-8 and LDH assays showing that Gpx4 shRNA reversed the protective effects of DGAT1 inhibition in OGD/R-treated highly differentiated PC12 cells. **G** Optical microscopy showing that Gpx4 shRNA reversed the improvements in cell morphology and number induced by DGAT1 inhibition in OGD/R-treated highly differentiated PC12 cells

As shown in Fig. 4A, OGD/R-treated highly differentiated PC12 cells displayed pronounced ferroptosis-related mitochondrial changes under TEM, including swelling, cristae loss, reduced membrane integrity, and outer membrane rupture, compared to the control group. Treatment with the DGAT1 inhibitor A 922500 significantly mitigated these mitochondrial alterations. Subsequently, western blot analysis indicated that both Fsp1 and Gpx4 levels markedly decreased in OGD/R-treated PC12 cells (Fig. 4B). DGAT1 inhibition significantly reversed the reduction of Gpx4, but had no significant effect on Fsp1 (Fig. 4B). Next, highly differentiated PC12 cells were infected with lentivirus carrying Gpx4 shRNA (Fig. 4C), and LV-956 was identified as the most effective sequence for knocking down Gpx4 expression (Fig. 4D), which was used in subsequent experiments. As shown in Fig. 4E, TEM revealed that Gpx4 shRNA significantly aggravated ferroptosis-related mitochondrial changes, such as swelling, cristae loss, and outer membrane rupture, reversing the protective effects of DGAT1 inhibition. As shown in Fig. 4F, Gpx4 shRNA significantly decreased the elevated cell viability induced by DGAT1 inhibition, and reversed the reduction in LDH levels caused by DGAT1 inhibition. The optical microscope observation, as shown in Fig. 4G, indicated that the highly differentiated PC12 cells in the OGD/R group exhibited significant damage, including cell shrinkage, deformation, disorganized structure, and a reduced number of cells. These morphological abnormalities were significantly improved after DGAT1 was inhibited, but this improvement was notably reversed by Gpx4 shRNA (Fig. 4G). These results indicated that DGAT1 inhibition suppressed ferroptosis by upregulating Gpx4, thereby alleviating cell damage in highly differentiated PC12 cells caused by OGD/R.

### Inhibition of DGAT1 mitigated brain neurological impairment in MCAO rats by attenuating ferroptosis through upregulating Gpx4

**Fig. 5:**
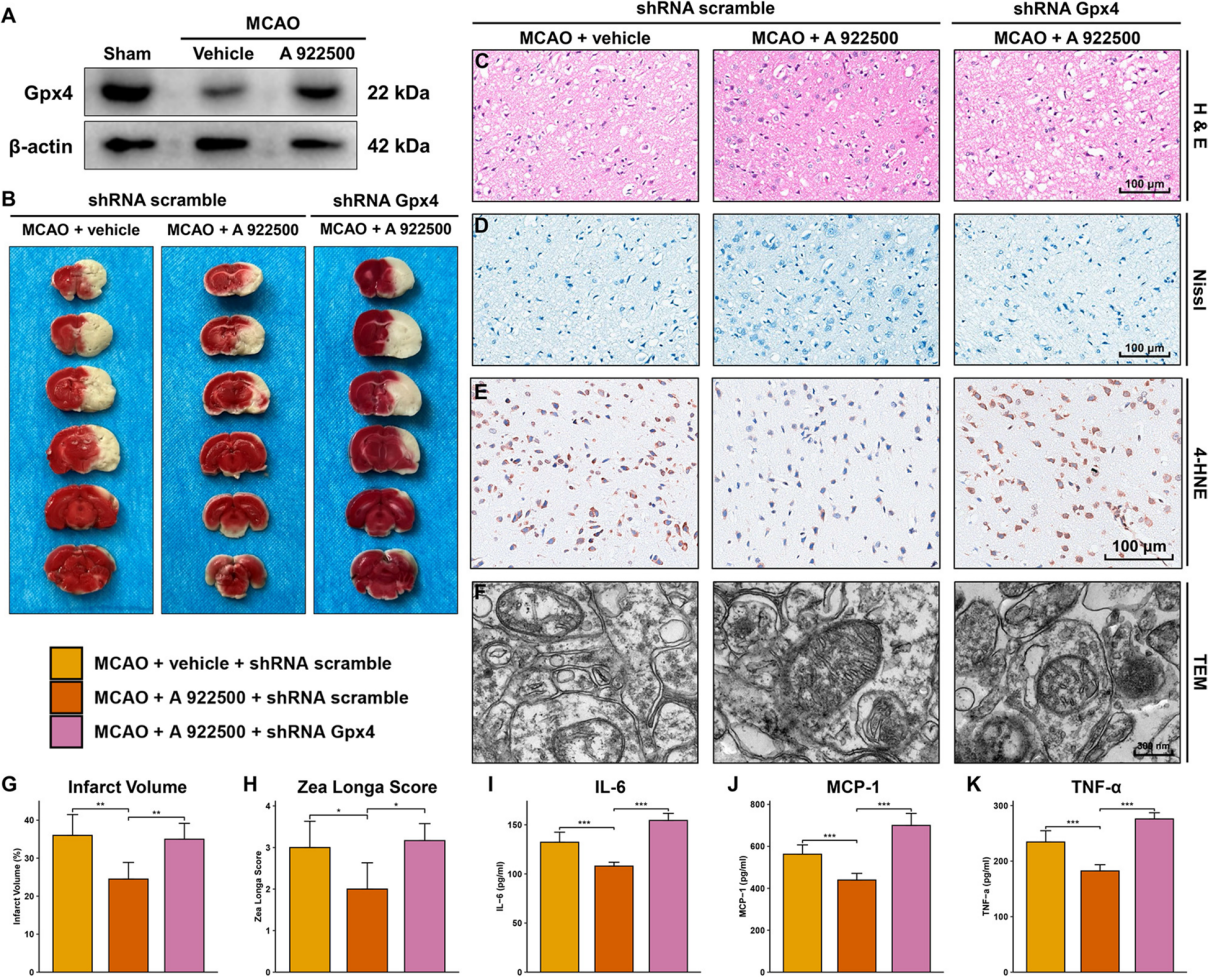
DGAT1 inhibition mitigates neurological impairment in MCAO rats by attenuating ferroptosis through upregulating Gpx4. **A** Western blot analysis showing that DGAT1 inhibition upregulated Gpx4 expression, which was decreased in MCAO rats. **B** TTC staining analysis showing that the reduction in infarct volume caused by DGAT1 inhibition was reversed by Gpx4 shRNA. **C** H&E staining showing that Gpx4 shRNA reversed the protective effects of DGAT1 inhibition on cerebral tissue damage in MCAO rats. **D** Nissl staining showed that Gpx4 shRNA reversed the protective effects of DGAT1 inhibition on neuronal injury in MCAO rats. **E** Gpx4 shRNA increased 4-HNE levels, which had been reduced by DGAT1 inhibition in the MCAO model. **F** TEM analysis showing that Gpx4 shRNA reversed the improvements in ferroptosis-related mitochondrial damage induced by DGAT1 inhibition. **G** Bar plot of TTC staining results showing that Gpx4 shRNA reversed the DGAT1 inhibition-induced reduction of infarct volume in MCAO rats. **H** Gpx4 shRNA reversed the improvement in Zea Longa neurological scores induced by DGAT1 inhibition. **I** Gpx4 shRNA increased IL-6 levels, which had been reduced by DGAT1 inhibition in the MCAO model. **J** Gpx4 shRNA increased MCP-1 levels, which had been reduced by DGAT1 inhibition in the MCAO model. **K** Gpx4 shRNA increased TNF-α levels, which had been reduced by DGAT1 inhibition in the MCAO model

As shown in Fig. 5A, Gpx4 was significantly downregulated in MCAO rats, while treatment with the DGAT1-specific inhibitor A 922500 significantly upregulated Gpx4 levels compared to the MCAO group. TTC staining demonstrated that the reduction in infarct volume induced by DGAT1 inhibition was reversed by pre-treatment with lentivirus carrying Gpx4 shRNA via intracerebroventricular injection (Fig. 5B, G). Similarly, H&E and Nissl staining showed that Gpx4 shRNA significantly reversed the protective effects of DGAT1 inhibition on cerebral tissue and neuronal injury in MCAO rats (Fig. 5C, D). Gpx4 shRNA significantly elevated 4-HNE levels compared to the MCAO + A 922500 group, reversing the lipid peroxidation reduction caused by DGAT1 inhibition (Fig. 5E). Additionally, TEM analysis showed that Gpx4 shRNA reversed the improvements in ferroptosis-related mitochondrial damage achieved by DGAT1 inhibition (Fig. 5F). Gpx4 knockdown also significantly reversed the improvement in Zea Longa neurological scores induced by DGAT1 inhibition (Fig. 5H). Furthermore, levels of the inflammatory factors IL-6, MCP-1, and TNF-α, which were reduced by DGAT1 inhibition, were significantly elevated following Gpx4 shRNA treatment (Fig. 5I, J, K). These results indicated that in MCAO rats, DGAT1 inhibition upregulated Gpx4, thereby downregulating 4-HNE, resisting ferroptosis, and ultimately exerting neuroprotective effects.

### Inhibition of DGAT1 protected highly differentiated PC12 Cells in the OGD/R model by resisting ferroptosis through improving mitochondrial function

**Fig. 6:**
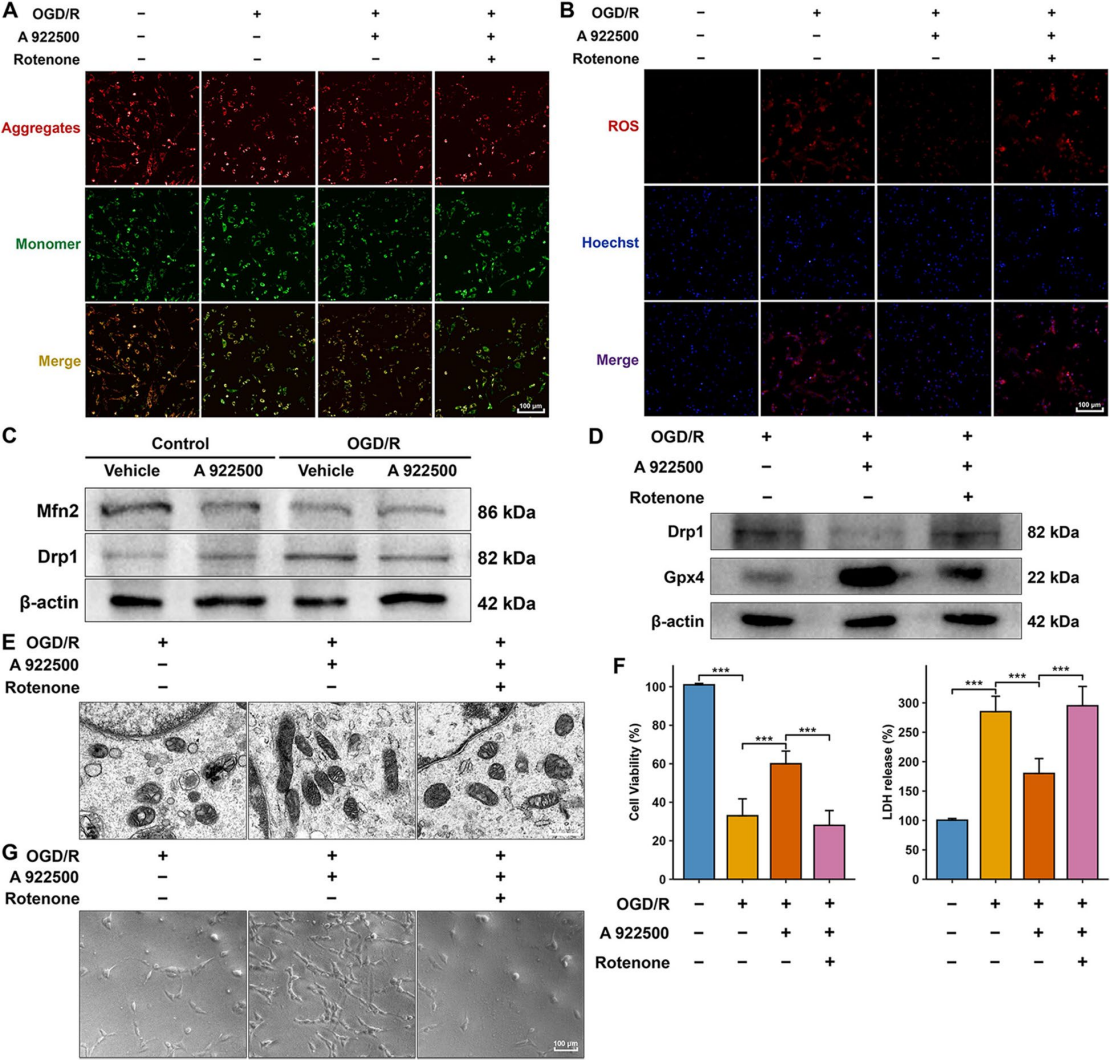
DGAT1 inhibition protects highly differentiated PC12 cells in the OGD/R model by resisting ferroptosis through improving mitochondrial function. **A** JC-1 assay showing the effect of DGAT1 inhibition on mitochondrial membrane potential in the OGD/R model. **B** ROS assay showing the effect of DGAT1 inhibition on reducing ROS levels in the OGD/R model. **C** Western blot analysis showing the effect of DGAT1 inhibition on mitochondrial fission (Drp1) and fusion (Mfn2) proteins in the OGD/R model. **D** Western blot analysis showing the influence of rotenone treatment (mitochondrial dysfunction) on Gpx4 expression in the OGD/R model. **E** TEM showing that rotenone (inhibitor of mitochondrial function) significantly reversed the protective effects of DGAT1 inhibition against ferroptosis-related mitochondrial damage in the OGD/R model. **F** CCK-8 and LDH assays showing that rotenone reduced cell viability and increased LDH release, reversing the protective effects of DGAT1 inhibition in the OGD/R model. **G** Optical microscopy images showing that DGAT1 inhibition improved cell morphology and number, and that these effects were reversed by rotenone in OGD/R-treated highly differentiated PC12 cells

Using the OGD/R model in highly differentiated PC12 cells, we further explored the upstream mechanism by which DGAT1 inhibition suppressed ferroptosis. The JC-1 assay demonstrated that OGD/R significantly decreased the mitochondrial membrane potential, while DGAT1 inhibition significantly increased it compared to the OGD/R group (Fig. 6A). Moreover, treatment with rotenone reversed the effect of DGAT1 inhibition on increasing mitochondrial membrane potential (Fig. 6A). As shown in Fig. 6B, OGD/R significantly increased ROS levels, whereas DGAT1 inhibition significantly reduced ROS levels. Similarly, rotenone reversed the effect of DGAT1 inhibition on ROS reduction (Fig. 6B). Western blot analysis indicated that OGD/R significantly reduced Mfn2 levels, suggesting decreased mitochondrial fusion, and significantly increased Drp1 levels, indicating enhanced mitochondrial fission (Fig. 6C). However, the DGAT1-specific inhibitor A 922500 reversed the OGD/R-induced increase in Drp1 levels but did not affect Mfn2 levels, suggesting that DGAT1 inhibition may exert protective effects in OGD/R by reducing mitochondrial fission (Fig. 6C). Similarly, in vivo experiments demonstrated that Drp1 expression was significantly elevated in MCAO rats compared to the sham group, and was markedly reduced following DGAT1 inhibition (Supplementary Figure). Figure 6D showed that rotenone significantly increased Drp1 levels, which had been reduced by DGAT1 inhibition, and significantly decreased Gpx4 levels, which had been elevated by DGAT1 inhibition. TEM indicated that after inhibiting mitochondrial function with rotenone, the protective effects of DGAT1 inhibition against ferroptosis-related mitochondrial damage in the OGD/R model were significantly reversed, as evidenced by mitochondrial cristae damage and shortened mitochondrial length (Fig. 6E). The CCK-8 assay demonstrated that rotenone significantly reduced the increased cell viability induced by DGAT1 inhibition in the OGD/R model, and elevated LDH levels that had been reduced by DGAT1 inhibition (Fig. 6F). Optical microscopy revealed that the beneficial effects of DGAT1 inhibition on cell morphology and number in highly differentiated PC12 cells were significantly reversed by rotenone treatment (Fig. 6G). These findings suggest that DGAT1 inhibition improves mitochondrial function, thereby upregulating Gpx4 and suppressing ferroptosis, ultimately protecting highly differentiated PC12 cells in the OGD/R model.

### Inhibition of DGAT1 improved mitochondrial function by upregulating β-Oxidation in the OGD/R model

**Fig. 7:**
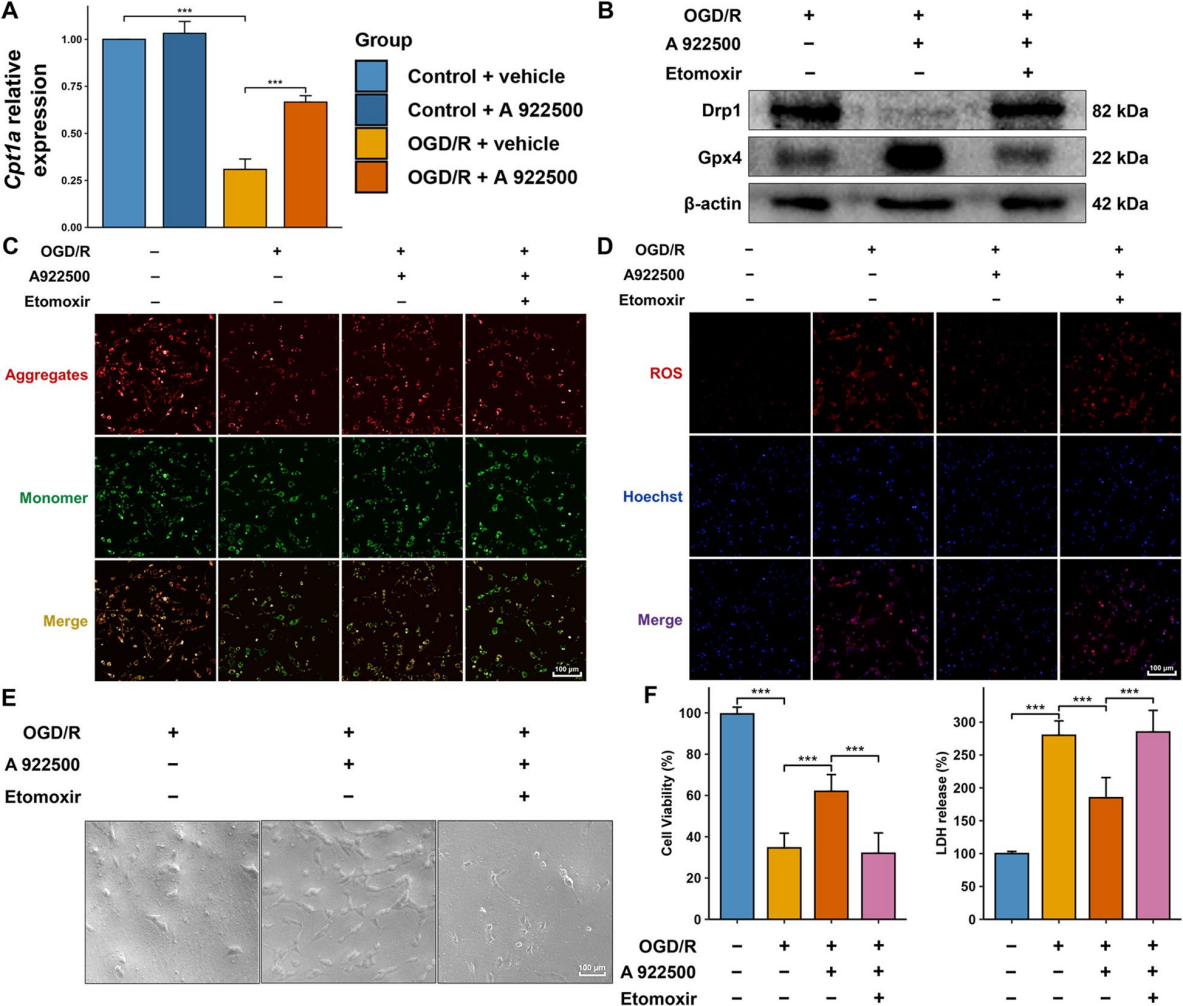
DGAT1 inhibition improves mitochondrial function by upregulating β-oxidation in the OGD/R model. **A** qPCR showing that DGAT1 inhibition increased *Cpt1a* mRNA expression compared to the OGD/R group. **B** Western blot analysis showing that the β-oxidation inhibition by etomoxir reversed the DGAT1 inhibition-induced decrease in Drp1 levels and the increase in Gpx4 levels in the OGD/R model. **C** JC-1 assay showing that β-oxidation inhibition decreased mitochondrial membrane potential, which had been improved by DGAT1 inhibition in the OGD/R model. **D** ROS assay showing that β-oxidation inhibition increased ROS levels, which had been reduced by DGAT1 inhibition in the OGD/R model. **E** Optical microscopy showing that the improvements in PC12 cell morphology and number caused by DGAT1 inhibition were reversed by β-oxidation inhibition in the OGD/R model. **F** CCK-8 and LDH assays showing that β-oxidation inhibition decreased cell viability and increased LDH levels, reversing the protective effects of DGAT1 inhibition in the OGD/R model

We speculated that in the OGD/R model, DGAT1 inhibition might improve mitochondrial function by enhancing β-oxidation. Using qPCR, we found that OGD/R significantly downregulated the mRNA expression of *Cpt1a* in highly differentiated PC12 cells, whereas DGAT1 inhibition led to a significant upregulation of *Cpt1a* compared to the OGD/R group (Fig. 7A), suggesting that DGAT1 inhibition upregulated β-oxidation in the OGD/R model. Western blot analysis showed that in the OGD/R model, the β-oxidation inhibitor etomoxir reversed the DGAT1 inhibition-induced decrease in Drp1 levels and reduced the DGAT1 inhibition-induced increase in Gpx4 levels (Fig. 7B). The JC-1 assay indicated that β-oxidation inhibition worsened the mitochondrial function improvements induced by DGAT1 inhibition in the OGD/R model (Fig. 7C). The ROS assay demonstrated that β-oxidation inhibition increased the ROS levels that had been reduced by DGAT1 inhibition in the OGD/R model (Fig. 7D). Optical microscopy revealed that the improvements in PC12 cell morphology and number induced by DGAT1 inhibition were reversed by β-oxidation inhibition in the OGD/R model (Fig. 7E). Additionally, the increased cell viability induced by DGAT1 inhibition was reduced by etomoxir treatment, while the decreased LDH levels were elevated by etomoxir treatment. These findings suggest that in the OGD/R model, DGAT1 inhibition exerts protective effects by enhancing mitochondrial function through increased β-oxidation, which in turn upregulates Gpx4 expression and suppresses ferroptosis.

## Discussion

This study represents the first investigation into the potential role of DGAT1 in ischemic stroke. In vivo experiments showed that DGAT1 inhibition improved neurological scores, reduced infarct volume, and alleviated brain tissue damage and neuronal injury in MCAO rats. In vitro experiments demonstrated that DGAT1 inhibition enhanced cell viability and reduced LDH levels in OGD/R-treated highly differentiated PC12 cells. Further investigations revealed that DGAT1 inhibition exerts its protective effects in both the MCAO and OGD/R models by suppressing ferroptosis via upregulating Gpx4. Finally, in vitro experiments indicated that the enhanced β-oxidation and improved mitochondrial function were involved in the upstream mechanisms through which DGAT1 inhibition exerted its protective effects by upregulating Gpx4 and inhibiting ferroptosis.

As a key enzyme in the synthesis of TAG, DGAT1 inhibition inevitably impacts lipid metabolism. The important role of lipid metabolism in ischemic stroke has been clearly established (Kloska et al. 2020), and targeting metabolic reprogramming may offer a novel approach to alleviating neuronal damage in ischemic stroke (Liang et al. 2021). Recent studies have increasingly shown that abnormal lipid metabolism is a crucial pathological mechanism in the progression of ischemic stroke (Bernoud-Hubac et al. 2024), and DGAT1 is a promising candidate as a key regulatory molecule in this process. Here, in vitro experiments demonstrated that OGD/R caused a significant downregulation of *Cpt1a* mRNA expression, which was consistent with previous findings by Rau et al. (Rau et al. 2012). In fact, DGAT1 deficiency has been shown to enhance the expression of genes involved in fatty acid oxidation while reducing the expression of genes related to lipid synthesis (Chen et al. 2002). CPT1 A catalyzes the transfer of the acyl group from long-chain fatty acyl-CoA to carnitine, facilitating its uptake by mitochondria for β-oxidation (Wolfgang et al. 2006). Under hypoxic conditions, enhanced glycolysis typically downregulates other energy production pathways, including fatty acid β-oxidation. This aligns with the upregulation of DGAT1 observed in both MCAO and OGD/R models, as DGAT1 upregulation promotes the conversion of fatty acids into triglycerides, further reducing β-oxidation. Our in vivo experiments indicated that DGAT1 inhibition led to upregulated *Cpt1a* expression in OGD/R-treated PC12 cells, suggesting a potential enhancement in β-oxidation. Furthermore, our in vitro experiments demonstrated that the β-oxidation inhibitor etomoxir reversed the protective effects of DGAT1 inhibition in OGD/R-treated PC12 cells, providing further evidence that the beneficial effects of DGAT1 inhibition are mediated through enhanced β-oxidation.

Although the brain makes up only 2% of body weight, it consumes about 20% of the body's oxygen, reflecting its heavy dependence on mitochondrial activity for energy and normal function (Erecińska and Silver 2001). Mitochondrial dysfunction after ischemic stroke, caused by electron transport chain damage and disrupted ATP production and oxidative phosphorylation, leads to excessive ROS production, mitochondrial swelling, and membrane rupture, further exacerbating neuronal damage (Andrabi et al. 2020; Bakthavachalam and Shanmugam 2017). The electron transport chain generates ROS as a byproduct during normal cellular respiration, particularly from Complex I and Complex III, where electrons can leak and react with oxygen to form superoxide radicals (Bae et al. 2011; Brand 2016). In ischemic conditions, the electron transport chain is impaired, leading to an increase in electron leakage and ROS overproduction, which damages mitochondrial lipids, proteins, and mtDNA, thereby disrupting mitochondrial membrane integrity and contributing to mitochondrial swelling and rupture (Chen et al. 2008). Increased ROS levels contribute to the activation of the mitochondrial fission protein Drp1, promoting mitochondrial fragmentation in response to oxidative stress (Wu et al. 2011). This fragmentation worsens mitochondrial dysfunction by disrupting morphology and function, creating a vicious cycle of oxidative stress and mitochondrial damage (Zhou et al. 2017). Our in vitro experiments revealed that DGAT1 inhibition improved mitochondrial function in the OGD/R model, as evidenced by increased mitochondrial membrane potential, decreased ROS release, and reduced expression of the mitochondrial fission protein Drp1. Additionally, we observed that co-treatment with the mitochondrial inhibitor rotenone significantly reversed the protective effects of DGAT1 inhibition, leading to cell morphology deterioration, decreased viability, and increased LDH levels, further confirming the central role of mitochondrial improvement in mediating the effects of DGAT1 inhibition. Additionally, we hypothesize that the upregulation of β-oxidation following DGAT1 inhibition increases acetyl-CoA production, enhancing its entry into the tricarboxylic acid (TCA) cycle, thereby promoting ATP production and further improving mitochondrial function (Bartlett and Eaton 2004). This hypothesis was supported by experiments showing that the β-oxidation inhibitor etomoxir counteracted the mitochondrial improvements induced by DGAT1 inhibition in the OGD/R model, indicating that its protective effects were mediated through enhanced β-oxidation.

Ferroptosis is a novel form of programmed cell death, characterized by the accumulation of lipid peroxides and ROS (Yang and Stockwell 2016). The morphological characteristics of ferroptosis is mitochondrial shrinkage, cristae reduction or loss, and outer membrane rupture or damage (Chen et al. 2021a). While numerous studies have explored the role of ferroptosis in cancer (Lei et al. 2022; Chen et al. 2021b), recent research has increasingly highlighted its involvement in the progression of ischemic stroke (Bu et al. 2021; Liu et al. 2022b). The ferroptosis-specific inhibitor ferrostatin-1 has been reported to alleviate ischemic injury in MCAO rats (Liu et al. 2023a), further highlighting the critical role of ferroptosis in the progression of ischemic stroke. GPX4 is a selenoprotein that uses glutathione (GSH) as a cofactor to reduce toxic lipid peroxides into non-toxic lipid alcohols, thus preventing ferroptosis (Seibt et al. 2019). In ischemic stroke models, GPX4 expression is notably downregulated due to several interrelated factors, primarily oxidative stress, depletion of GSH, and disruption of energy metabolism, limiting its protective function (Alim et al. 2019). Numerous previous studies have reported that various compounds and natural products regulate GPX4-dependent ferroptosis in MCAO and OGD/R models, contributing to the alleviation of hypoxic injury (Bartlett and Eaton 2004; Yang and Stockwell 2016; Chen et al. 2021a, 2021b; Lei et al. 2022; Bu et al. 2021; Liu et al. 2022b; Liu et al. 2023b; Seibt et al. 2019; Alim et al. 2019; Fan et al. 2023; Li et al. 2022; Li et al. 2024; Zhu et al. 2024; Zhang et al. 2024; Yang et al. 2022). Our study demonstrated that DGAT1 inhibition suppressed ferroptosis in MCAO rats, as indicated by the restoration of mitochondrial morphology, downregulation of lipid peroxidation markers 4-HNE and MDA, increased SOD levels, and reduced levels of inflammatory factors. Additionally, in vitro experiments showed that DGAT1 inhibition enhanced β-oxidation and mitochondrial function, increasing mitochondrial membrane potential and reducing ROS. This likely reduced Gpx4 consumption, upregulated its expression, and downregulated the lipid peroxidation marker 4-HNE, ultimately suppressing ferroptosis. Furthermore, the protective effects of DGAT1 were significantly diminished in both in vivo and in vitro experiments when Gpx4 was knocked down using shRNA, indicating that DGAT1 inhibition exerts beneficial effects in MCAO and OGD/R models by suppressing ferroptosis. In summary, our study reveals that DGAT1 inhibition upregulates Cpt1a expression, promotes fatty acid β-oxidation, and improves mitochondrial membrane potential while reducing ROS production. These mitochondrial improvements contribute to increased GPX4 expression and reduced lipid peroxidation, thereby suppressing ferroptosis and ultimately protecting against ischemic injury.

The findings of this study have potential clinical relevance. Specifically, in addition to standard thrombolytic therapy, DGAT1 inhibition may represent a promising neuroprotective approach, particularly in patients with elevated DGAT1 expression. This could pave the way for personalized therapeutic strategies in ischemic stroke management. Nevertheless, translating these findings into clinical application entails several challenges. Species-specific pharmacokinetic and pharmacodynamic differences, as well as the identification of optimal dosing regimens, must be carefully considered. Furthermore, the development of targeted drug delivery systems may enhance therapeutic efficacy by enabling the selective transport of DGAT1 inhibitors to ischemic brain regions, thereby minimizing off-target effects. Future studies should also include lipidomics analyses to identify key lipid species and elucidate the specific mechanisms by which DGAT1 inhibition modulates ferroptosis and attenuates ischemic injury. Finally, the long-term effects of DGAT1 inhibition on neuroplasticity and post-stroke functional recovery remain important topics for future investigation.

## Conclusions

In summary, our study suggests that DGAT1 inhibition may provide neuroprotection in ischemic stroke by upregulating β-oxidation, improving mitochondrial function, and suppressing ferroptosis.

## Supplementary Information

Supplementary Material 1.

## Data Availability

Data sharing not applicable to this article as no datasets were generated or analyzed during the current study.

## Abbreviations

tPA: Tissue plasminogen activator
TAG: Triacylglycerol
DGAT1: Diacylglycerol O-acyltransferase 1
DAG: Diacylglycerol
ROS: Reactive oxygen species
MCAO: Middle cerebral artery occlusion
OGD/R: Oxygen–glucose deprivation/reperfusion
GEO: Gene Expression Omnibus
SD: Sprague–Dawley
SPF: Specific pathogen-free
CCA: Common carotid artery
ICA: Internal carotid artery
ECA: External carotid artery
IHC: Immunohistochemistry
TSA: Tyramide Signal Amplification
NGF: Nerve growth factor
FBS: Fetal bovine serum
BCA: Bicinchoninic Acid
PVDF: Polyvinylidene fluoride
TTC: 2,3,5-Triphenyltetrazolium chloride
HE: Hematoxylin–Eosin
CCK-8: Cell Counting Kit-8
OD: Optical density
LDH: Lactate dehydrogenase
PB: Phosphate buffer
TEM: Transmission electron microscope
SOD: Superoxide dismutase
MDA: Malondialdehyde
ELISA: Enzyme-Linked Immunosorbent Assay
MOI: Multiplicity of infection
DHE: Dihydroethidium
qRT-PCR: Quantitative reverse transcription PCR
TCA: Tricarboxylic acid,
GSH: Glutathione
