# Supplementary material for: Inhibition of diacylglycerol O-acyltransferase 1 provides neuroprotection by inhibiting ferroptosis in ischemic stroke
Source: Mol Med. 2025 May 15;31:191. doi: 10.1186/s10020-025-01255-w (PMC12082899; doi:10.1186/s10020-025-01255-w)
Supplement: Supplementary file 1 — Supplementary Material 1. [file 10020_2025_1255_MOESM1_ESM.pdf]

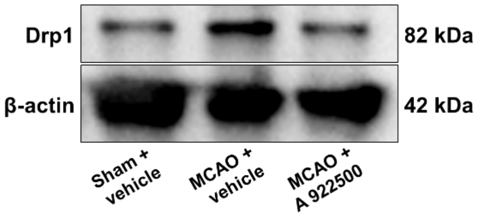

Supplementary Fig. Western blot analysis showing increased Drp1 levels in the MCAO group compared to the sham group, which were significantly reduced following treatment with the DGAT1 inhibitor A 922500.
